# Women’s mental healthcare in the Republic of Ireland

**DOI:** 10.1192/bji.2025.10072

**Published:** 2026-05

**Authors:** Yvonne Hartnett, Siobhan MacHale, Richard Duffy

**Affiliations:** 1 MRCPsych, PhD Candidate, School of Medicine, University College Dublin, Dublin, Ireland Email: hartnety@tcd.ie; 2 FRCPsych, Consultant Psychiatrist, Department of Liaison Psychiatry, Beaumont Hospital, Dublin, Ireland; 3 MRCPsych, Consultant Psychiatrist, Perinatal Mental Health Service, Rotunda Hospital, Dublin, Ireland

**Keywords:** Women’s mental health, asylums, history of psychiatry, psychiatric services, perinatal psychiatry

## Abstract

This paper describes recent developments in mental healthcare for women in Ireland and sets them in their historical context. The treatment of women’s mental health in the Republic of Ireland has evolved drastically since the 19th century, when institutions such as ‘lunatic asylums’ and Magdalene Laundries were commonplace. With deinstitutionalisation in the late 20th century, services adapted to community models, though these have remained underfunded. Recent years have witnessed dramatic developments in female-specific services and policies. Despite this progress, unmet needs remain in eating disorders, mental health support for pregnancy loss, infertility and embedding trauma-informed care.

The Republic of Ireland has taken a number of steps in the last decade to improve women’s mental health. This paper provides historical context for these advances, describes the advances themselves and identifies areas requiring further development. Most mental healthcare in Ireland is provided publicly through community mental health teams; 46% of Ireland’s 5.4 million population have private health insurance and can access private or public services. Specific services exist for multiple specialities, including child and adolescent, addiction and perinatal psychiatry; services are currently being rolled out for eating disorders and adult attention-deficit hyperactive disorder. Counselling services, services for domestic violence and sexual trauma sit outside of formal mental health services.

## The age of institutions

The Republic of Ireland (as part of the United Kingdom until 1921) established its asylum system before Great Britain.^1^ Ireland’s first asylum, the Richmond Lunatic Asylum, opened in 1814; similar systems were not seen in England until 1845 and Scotland until 1857.^1,2^ The growth of asylums accelerated with the Lunacy (Ireland) Act of 1821 and continued until the late 20th century, peaking in the 1950s, when Ireland had the highest per capita population in psychiatric institutions globally, with nearly 20 000 in-patients.^3,4^

The primary function of these institutions was to uphold public order rather than provide treatment. They served to segregate not only individuals with mental illnesses but also those who were non-conforming or socially deviant.^2^ Many societal factors made women particularly vulnerable to these practices, including financial dependence on families, a lack of resources for women who were intellectually disabled or unmarried mothers, stigma associated with mental illnesses and a belief that women were more prone to mental illness.^6^ While healthcare in Ireland was often provided by the religious orders, this was not the case with mental illness; the evolution of the asylums was led by the government, medical profession, Houses of Industry and prisons.^1,2^ The Dangerous Lunatics Act of 1838 associated mental illness with criminality and provisions allowed alleged safety concerns to facilitate admission to asylums, which was often used to admit women.^1^

For some women, particularly those with pregnancy-related symptoms, admissions were a place of reprieve from repeated pregnancies and nursing infants.^2^ These women were prescribed rest and nutrition; once they were physically stronger, they were often discharged.^2^ However, many women were less fortunate and became long-term residents of the asylums, some even going on to work there as staff.^1,2^

Dr Ada English (1875–1944) was one of the first female psychiatrists, in Ireland and a key reformer.^1^ As an elected member of the second Dáil (Irish parliament), her campaigning helped shape The Mental Treatment Act of 1945, which introduced the concept of community care. Over 30 years later, in 1984, a report on Irish psychiatric services demonstrated how slowly deinstitutionalisation was progressing. It concluded: ‘there must be a decided shift in the pattern of care from an institutional to a community setting’.^5^ This focus on in-patient care disproportionately impacted women, as the report noted that the prevalence of mental illness was twice as high in females; however, admission rates were higher in men.

During the 19th century and 20th century, asylums were not the sole institutions where women were unjustly detained in Ireland. In parallel with the expansion of the asylum system, the ‘Magdalene Laundries’ were established and primarily run by religious congregations.^6^ The complex political and cultural background to these laundries and their impact are excellently discussed by McGettrick et al.^6^ These facilities housed ‘fallen women,’ whose behaviour deviated from so called proper behaviour, usually women who became pregnant outside marriage. Many women incarcerated there suffered with mental illness and were already vulnerable. They were confined to the laundries, deprived of their liberty for indefinite periods, subject to unpaid labour, separated from their children and isolated from society. The last laundry closed in 1996. As these laundries were outside of the asylum system, they were not subject to regular inspection as the asylums were.

## Mental health provision and policy in the 21st century

Rapid deinstitutionalisation in the late 20th century saw the closure of the asylums and laundries. The community services proposed to replace them have remained underfunded, with mental health services accounting for only 6% of the national health budget in comparison with 12% in similar countries.^3^ The need to accommodate the differing health needs and barriers faced by women was highlighted by the Women’s Health Council as early as 2005.^7^ But progress enacting their recommendations has been slow.

Political will to improve healthcare quality and experiences for women found new momentum following a national scandal involving inaccuracies in cervical screening.^8^ The resulting inquiry by Dr Gabriel Scally highlighted persistent misogyny and paternalism in Irish healthcare.

The establishment of the Women’s Health Taskforce and Women’s Health Fund has ensured a rapid increase in funding for women-specific health services for both physical and mental healthcare. Since 2022, women’s mental health has been embedded within national mental health policy, and a charter for women’s mental health has been established comprising three tenets: (a) gender awareness in delivery of care, (b) a trauma-aware approach and (c) the systemic collection of data on sociodemographic risk factors relating to the marginalisation of women.^9^

## Specialist perinatal mental health services

A major shift in women’s mental health in the Republic of Ireland in recent years has been the development of perinatal mental health services. In 2017, there were fewer than four psychiatrists with dedicated perinatal roles nationally.^10^ The driving force for this change came through the National Maternity Strategy (Ireland) 2016–2026, rather than through mental health policy and planning.^11^

In 2017, the Irish Health Service Executive published the Specialist Perinatal Mental Health Services Model of Care (MOC).^10^ Funding came promptly on the back of the model, and the roll-out drastically reformed services nationally.^11^ The MOC created a hub and spoke model with full multidisciplinary teams located at the six largest hub sites and mental health midwives in spoke sites.^10^ There are now nearly 100 staff working in perinatal mental health in Ireland. The rapid expansion was aided by the development of training opportunities. Perinatal psychiatry has been a higher training specialisation option in the College of Psychiatrists of Ireland since 2019; the College also established a perinatal special interest group in 2023. Two universities developed courses in perinatal mental health. National-level data collection has been implemented through collaboration with the National Perinatal Epidemiology Centre.^11^

The MOC has its limitations. Its implementation has not been universally successful, and there are areas for expansion in future revisions.^11^ First, Ireland is still without a mother and baby unit (MBU). Second, the level of multidisciplinary care individuals receive at spoke sites is highly limited, creating disadvantages for women in rural communities. A revision of the 2017 MOC is currently underway. Future iterations of the model will hopefully give greater consideration to fertility treatment, infant mental health, paternal mental health and bereavement. Ireland has much to learn from countries like Scotland and New Zealand, which are also attempting to address the challenges of low population density on a similar scale.

## Mental health grounds for termination of pregnancy

Ireland’s termination of pregnancy legislation was recently revised with the Health (Regulation of Termination of Pregnancy) Act 2018, enacted in 2019. The passage of this legislation required a constitutional amendment, which necessitated a referendum.^12^ This expanded the circumstances in which a termination could be carried out and included provision for harm to mental health. Prior to this, terminations were only possible if an individual’s life was in danger: risk to health alone was insufficient.

The 2018 Act has explicitly defined health as physical or mental health. Termination is now possible up to viability where there is a serious risk of harm to health and where two medical practitioners agree that a termination will avert the risk. This is a higher bar than the English legislation, where the clinicians must be satisfied that continuance of the pregnancy would involve risk greater than if the pregnancy were terminated. While there are still many challenges concerning the legislation’s implementation and debate about its content, individuals whose mental health is harmed by pregnancy now have more options.^12^

## Future directions

Ireland has come an extraordinarily long way in recent years, but much work remains to be done in ensuring women’s mental health services are sufficiently resourced. Presently, there are only three public in-patient beds for eating disorders, and there are virtually no public services for adults seeking a diagnosis of autism. These are deficits that disproportionally affect women.^9^ The long-promised MBU is far from being established.^11^ Public fertility treatment is being rolled out with limited considerations of mental health needs. There is also a pressing need to introduce trauma-informed care throughout healthcare settings, including mental health, maternity and gynaecology settings.

The rapid expansion of perinatal mental health services demonstrates what is possible within a relatively short period of time, when there is broad buy-in from a wide range of stakeholders, including patients, advocacy groups, maternity services, politicians, colleges, training and professional bodies, and researchers.

## Data Availability

Data availability is not applicable to this article as no new data were created or analysed in this study.
